# Does ligation of the dorsal branch of the cephalic vein affect the patency of a distal forearm arteriovenous fistula? A randomised study

**DOI:** 10.1186/s12882-020-01823-8

**Published:** 2020-04-29

**Authors:** Hongtao Zhang, Guizhen Zhu, Lei Yan, Yang Lu, Fengmin Shao

**Affiliations:** 1 Blood Purification Center, The People’s Hospital of Zhengzhou University, Zhengzhou, China; 2 Blood Purification Center, Henan Provincial People’s Hospital, Zhengzhou, China; 3 Institute of Nephrolog, Zhengzhou, Henan China; 4 Department of Nephrology Henan Provincial People’s Hospital, Zhengzhou, China

**Keywords:** Cephalic vein, Radiocephalic arteriovenous fistula, Patency rate, Randomized controlled trial

## Abstract

**Background:**

Whether ligation of the dorsal branch of the cephalic vein during the surgical establishment of the radiocephalic arteriovenous fistula (RCAVF) favorably or adversely affects the patency rate of the RCAVF remains controversial. We performed a randomized controlled trial to evaluate the effect of dorsal branch ligation on the patency rate of RCAVF.

**Methods:**

A total of 115 patients who underwent surgical establishment were randomized to two groups treated with or without ligation of the dorsal branch of the cephalic vein during the surgical process. The primary patency rates of the RCAVF at 90, 270, and 360 days after the surgery and the secondary patency rates during a follow-up up to 1 year were compared.

**Results:**

The patency rate did not differ significantly between the two groups at 3, 9, or 12 months after the procedure (*P* > 0.05). The combined primary patency rates of the RCAVF in patients from both groups at 3, 9 and 12 months after the procedure were 87.6, 82, and 74.5% respectively, while the combined secondary patency rate was 92.2% at the 1-year follow-up. The Log-rank test indicated that the initial patency rate and secondary patency rate did not differ significantly between the two groups (*P* = 0.674 and 0.759, respectively).

**Conclusion:**

This clinical study indicated that ligation of the dorsal branch of the cephalic vein does not significantly affect the patency of the arteriovenous fistula with a 1-year follow-up.

**Trial registration:**

ISRCTN ISRCTN12288675, Registered 25 September 2019 in the ISRCTN registry. retrospectively registered.

## Background

Preserved function of vascular access is important for maintaining effective hemodialysis. The radiocephalic arteriovenous fistula (RCAVF), which was first reported in 1966 by Brescia and Cimino, has become the most commonly performed procedure for establishing vascular access in patients receiving hemodialysis, based on its high patency rate and low incidence of complications [1]. Clinically, a distal fistula is routinely established to provide additional puncture sites. Anatomically, an important dorsal branch of the cephalic vein can be found 2–3 cm above the wrist. It has been suggested that ligation of the dorsal branch of the cephalic vein during the surgical process of RCAVF establishment may improve its patency rate, because inevitably, hemodynamic abnormalities in the bifurcation of the dorsal cephalic branch site exist, which may increase the risk of stenosis of the RCAVF [2]. However, this is challenged by the hypothesis that the dorsal branch of the cephalic vein could serve as a spare channel when the trunk is occluded, and the patency of the dorsal branch could at least partly compensate the blood flow by the formation of collateral circulation [3], which may contribute to the patency of the RCAVF.

However, whether ligation of the dorsal branch of the cephalic vein actually affects the patency of the RCAVF has not been evaluated in a clinical trial. Therefore, in this randomized controlled trial, we aimed to compare the patency of the RCAVF in patients with and without ligation of the dorsal branch of the cephalic vein during the surgical processes.

## Methods

### Patients and study design

Patients who underwent a first-time surgical procedure for the induction of a RCAVF at Zhengzhou University People’s Hospital from January 2014 to January 2016 were included. And RCAVF location was determined with preference at the most distal possible site in the non-dominant arm. The inclusion criteria were as the follows: (1) existence of a cephalic vein dorsal branch was confirmed via vein mapping before the procedure; (2) the feasibility of the surgical procedure was confirmed by experienced surgeons via clinical assessment of the arterial pulse, expansion of the vein after blocking the proximal blood flow with a tourniquet, and bilateral arm blood pressure. Moreover, bilateral vascular assessment, including vascular diameters and arterial blood flow, was preformed by duplex ultrasound. and (3) negative findings were obtained on Allen’s test preoperatively. Patients without a cephalic vein dorsal branch or who were unable to provide informed consent were excluded. The protocol of the study was approved by the Ethics Committee of Zhengzhou University People’s Hospital before the enrollment of patients. All patients included in were randomly divided into two groups by sealed-envelope randomization, with about 1:1 ratio. And the envelopes were prepared by another clinician in advance. The baseline characteristics of the included patients were collected, including age, sex, primary disease, fistula location, vascular diameters, and puncture time of the fistula.

### Surgical establishment of a RCAVF

**Fig. 1:**
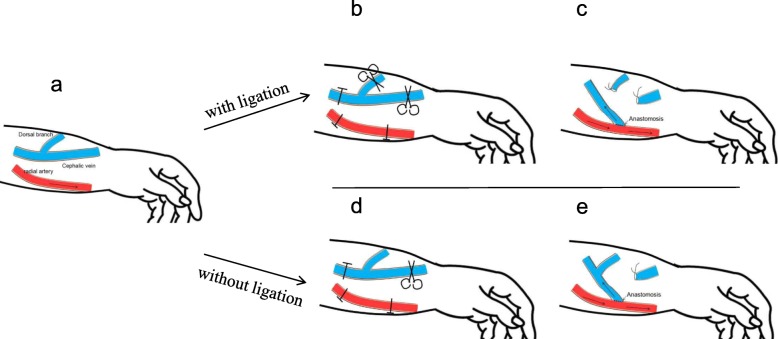
Creation of a radiocephalic arteriovenous fistula. **a** Separation of cephalic vein and radial artery; **b** ligation of distal cephalic vein and the dorsal branch with a 4–0 silk; **c** End-to-side anastomosis with a 7–0 silk; d, ligation of distal cephalic vein with a 4–0 silk; e, End-to-side anastomosis with a 7–0 silk

All the surgical process of the establishment of a RCAVF were performed by an experienced surgeon with patients under local anesthesia and receiving 7–0 end-to-side anastomosis. In those allocated to the ligation group, the dorsal branch of the cephalic vein was ligated with a 4–0 silk, while ligation of the dorsal branch of e vein was not performed in those allocated to the other group (Fig. 1).

### Follow-up and definitions of outcomes

The primary outcomes of the study were the primary patency rate of the RCAVF of patients in both groups at 3, 9, and 12 months after the surgical procedure. The primary patency rate refers to the time from the establishment of the fistula to the initial percutaneous transluminal angioplasty (PTA) or application of an intervention method to maintain the fistula patency. The definition of secondary patency rate was defined as the time from fistula establishment to fistula abandonment, or thrombosis needing a re-establishment of a new fistula [4]. When adverse events such as death, kidney transplantation, and switch to peritoneal dialysis occurred, the follow-up was considered complete.

### Statistical analysis

SPSS software (version 19.0) was used for statistical analyses. Quantitative and qualitative data were compared between both groups using Log-rank tests. The Kaplan-Meier survival analysis was performed to evaluate the differences in primary and secondary patency rates of the patients treated with an arteriovenous fistula with and without ligation of the dorsal branch (*P* < 0.05).

## Results

A total of 115 patients who underwent the surgical establishment of a distal autologous arteriovenous fistula were included. There were no statistically significant differences in age, sex, etiology of renal failure, artery and vein diameters, and puncture time between patients in the two groups **(**Table 1, both *P* > 0.05). The occurrence of swollen hand syndrome was observed only in the group without dorsal branch ligation. During the 1-year follow-up, two cases of death and one case of kidney transplantation occurred in the group with dorsal branch ligation, whereas one death and one case of switching to peritoneal dialysis occurred in the group without dorsal branch ligation.

**Table 1:** Baseline characteristics of the included patients

	With ligation (*n* = 58)	Without ligation (*n* = 57)	*P*-value
Age (years)	58 ± 52.76	57 ± 53.28	0.84
Gender (male/female)	30/28	31/26	0.85
Etiologies for kidney dysfunction			
Diabetes	14 (24.1%)	13 (22.8%)	0.946
Chronic nephritis	23 (39.7%)	24 (42.1%)	0.946
Hypertensive kidney damage	8 (13.8%)	6 (10.5%)	0.946
Others	13 (22.4%)	14 (24.6%)	0.496
Side (left/right)	42/16	35/22	0.238
Initial puncture time (days)	53 ± 39.08	55 ± 37.55	0.442
Artery diameters (millimeter)	2.08 ± 0.31	2.15 ± 0.45	0.401
Vein diameters (millimeter)	2.09 ± 0.58	2.16 ± 0.46	0.534
Swollen hand syndrome (%)	0	2 (3.5%)	0.153

There were no statistically significant differences between the two groups in the primary patency rates at 3, 9, and 12 months after the procedures (Table 2, both *P* > 0.05). The primary patency rates of the RCAVF of all patients in both groups at 3, 9 and 12 months after the procedure were 87.6, 82.0, and 74.5% respectively, while the combined secondary patency rate was 92.2% at the 1-year follow-up.

**Table 2:** Cumulative patency rate of the RCAVF in patients in the two groups

Follow-up time	With ligation	Without ligation	*P*-value
3 months	48/56(85.7%)	51/57(89.5%)	0.581
9 months	45/56(80.4%)	46/55(83.6%)	0.806
12 months	40/55(72.7%)	42/55(76.4%)	0.827

**Fig. 2:**
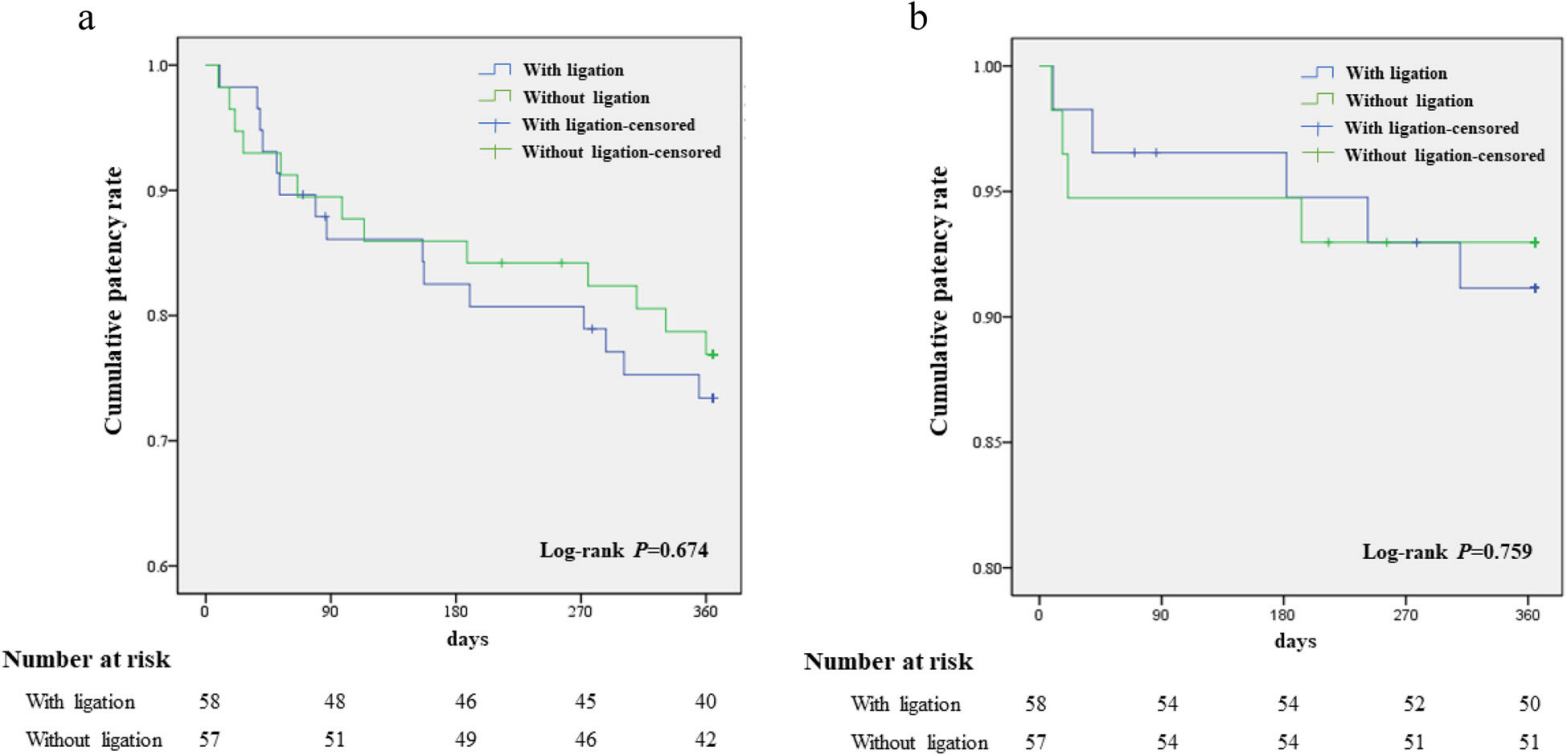
Kaplan-Meier survival analysis for evaluation of the differences in the primary and secondary patency rates in patients with and without ligation of the dorsal branch; **a** Kaplan-Meier survival analysis for the primary patency rate; **b** Kaplan-Meier survival analysis for the secondary patency rate

The Log-rank test indicated that the initial patency rate and secondary patency rate between the patients in the two groups were not statistically significant (*P* = 0.674 and 0.759, respectively; Fig. 2).

## Discussion

In this randomised study of Chinese patients with end-stage renal disease who underwent surgical establishment of a RCAVF, we found that ligation of the dorsal branch of the cephalic vein did not significantly affect the patency of the arteriovenous fistula within a 1-year follow-up.

A distal autologous fistula as a vascular access for patients with hemodialysis has many advantages, such as providing multiple puncture sites, reliably retaining a proximal arteriovenous access for a convenient way to correct failure of the fistula, and limited incidence rates of steal syndrome and heart failure [5]. However, the primary failure of the RCAVF is high, with documented primary patency rates of 58–63% according to a previous study [6, 7]. In the present study, the overall primary patency rate for patients in both groups was 74.5%. This may be because currently in China most patients receiving hemodialysis have chronic nephritis (accounts for 40.9% in this study), and the patients who began to undergo dialysis were relatively young (mean age, 55 years). It has been recognized that the diameter of preoperative blood vessels often affects the prognosis of the patients. Therefore, preoperative assessment is essential in clinical practice, preferably with vascular ultrasound examination rather than experience-based judgement of the surgeons.

The most common complication of an AVF is stenosis, which is also the main cause of poor vascular dysfunction or dysmaturity. A previous study that analyzed the initial failure of the RCAVF among 24 cases indicated that the dorsal branch of the cephalic vein was prone to progressive stenosis, which, from the authors’ point of views, may be related to the enhanced blood flow turbulence caused by the sheer force in the bifurcation of the vessels [8]. Furthermore, other researchers also believed that the dorsal branch of the cephalic vein plays an important role in the stenosis progression of the fistula outflow tract. They studied 57 patients who underwent first-time RCAVF establishment, and the dorsal cephalic branch was ligated or anastomosed according to the diameter and distance of the distal cephalic vein and the dorsal branch. The primary patency of the RCAVF in their cohort after 1 year was 69.2%, while the secondary patency rate was 87.9% after 1 year [2]. In our study, the primary and secondary patency rates after 1 year in patients with dorsal branch ligation were 72.7 and 92.2%, respectively. The differences may be explained by the different characteristics of the patients. In the previous study, 56.1% of the cohort had diabetes, versus 24.1% in our cohort [2]. Because diabetes is recognized as a major risk factor for stenosis, it is not surprising that the cohort of patients in the previous study had lower patency rates. Additionally, in another study including 52 cases of forearm fistula dysfunction and 17 cases of thrombosis formation in forearm fistula, the authors concluded that simple ligation or embolization of the branch cannot provide treatment for stenosis but may cause thrombosis or dysfunction [9]. Based on the findings of fistula radiography, except for those with excessive flows in the fistula, generally the collateral is difficult to develop. In fact, visualization of the dorsal branch under angiography may reveal the stenosis or occlusion in the proximal segment of the cephalic vein, and the dorsal branch may only be a reflection for shunting of the flow of blood that could not pass through the stenosis site [9]. A recent case report revealed successful resolution of trunk occlusion by puncturing the dorsal cephalic branch. In that case, a patient with chronic obstructive lesions near the anastomosis was treated with dorsal branch diversion and timely PTA, and subsequently the function of the fistula was restored [10]. In the present study, swollen hand syndrome was only observed in the group without dorsal branch ligation. Clinically, it appears as swelling of the hand. This venous hypertension is caused by occlusion of the central veins, reverse blood flow, and increased venous hydrostatic pressure, leading to swelling of the distal part of the limb. However, whether it is associated with the ligation with the dorsal branch of the hand remains to be determined. In addition, the dorsal branch of the cephalic vein could be used as a patch to successfully resolve stenosis in the immature fistula anastomosis [11]. Even if the proximal cephalic vein was lacked, but with good dorsal vein network, the efficiency of fistulas in the snuff-box region was excellent [12]. When the proximal cephalic vein was occluded, as a diversion channel, the dorsal cephalic branch was able to provide sufficient filling of the sequential veins and facilitate effective puncture if necessary [12]. However, our randomized study showed that the patency rates of the arteriovenous fistula with a 1-year follow-up did not differ significantly between those with and without ligation of the dorsal branch. These results demonstrate that the ligation of the dorsal branch of the cephalic vein does not significantly affect the patency of the arteriovenous fistula with a 1-year follow-up.

Our study has limitations. First, this was a small-scale, single-center study. The negative results may be due to the limited sample size included. Moreover, many factors not included in our analyses may affect the patency of the fistula. Finally, this was not a blinded trial, and the surgeons were aware of the grouping of the patients, which may introduce additional biases.

## Conclusion

In conclusion, the results of this clinical study indicate that ligation of the dorsal branch of the cephalic vein does not significantly affect the patency of the arteriovenous fistula with a 1-year follow-up. These results should be confirmed in large-scale trials.

## Data Availability

The datasets used and/or analysed during the current study available from the corresponding author on reasonable request.

## Abbreviations

AVF: Arteriovenous fistula
RCAVF: Radiocephalic arteriovenous fistula
PTA: Percutaneous transluminal angioplasty
